# Identification and Migration of Primordial Germ Cells in Atlantic Salmon, *Salmo salar*: Characterization of *Vasa*, *Dead End*, and *Lymphocyte Antigen 75* Genes

**DOI:** 10.1002/mrd.22142

**Published:** 2013-02-05

**Authors:** Kazue Nagasawa, Jorge MO Fernandes, Goro Yoshizaki, Misako Miwa, Igor Babiak

**Affiliations:** 1 Faculty of Biosciences and Aquaculture, University of NordlandBodø, Norway; 2 Department of Marine Biosciences, Tokyo University of Marine Science and TechnologyMinato-ku, Tokyo, Japan

## Abstract

No information exists on the identification of primordial germ cells (PGCs) in the super-order Protacanthopterygii, which includes the Salmonidae family and Atlantic salmon (*Salmo salar* L.), one of the most commercially important aquatic animals worldwide. In order to identify salmon PGCs, we cloned the full-length cDNA of *vasa*, *dead end* (*dnd*), and *lymphocyte antigen 75* (*ly75/CD205*) genes as germ cell marker candidates, and analyzed their expression patterns in both adult and embryonic stages of Atlantic salmon. Semi-quantitative RT-PCR results showed that salmon *vasa* and *dnd* were specifically expressed in testis and ovary, and *vasa*, *dnd*, and *ly75* mRNA were maternally deposited in the egg. *vasa* mRNA was consistently detected throughout embryogenesis while *dnd* and *ly75* mRNA were gradually degraded during cleavages. In situ analysis revealed the localization of *vasa* and *dnd* mRNA and Ly75 protein in PGCs of hatched larvae. Whole-mount in situ hybridization detected *vasa* mRNA during embryogenesis, showing a distribution pattern somewhat different to that of zebrafish; specifically, at mid-blastula stage, *vasa*-expressing cells were randomly distributed at the central part of blastodisc, and then they migrated to the presumptive region of embryonic shield. Therefore, the typical *vasa* localization pattern of four clusters during blastulation, as found in zebrafish, was not present in Atlantic salmon. In addition, salmon PGCs could be specifically labeled with a green fluorescence protein (GFP) using *gfp-rt-vasa* 3′-UTR RNA microinjection for further applications. These findings may assist in understanding PGC development not only in Atlantic salmon but also in other salmonids.

## INTRODUCTION

Development of primordial germ cells (PGCs) is fundamental to further gonad formation and affects individual fertility in vertebrates (Molyneaux and Wylie, 2004). In teleosts, it has been reported that morpholino knockdown of *dead end* (*dnd*) leads to subsequent PGC death due to the loss of function for normal migration and survival (Weidinger et al., 2003). The resulting PGC-ablated fish are then sterile (Slanchev et al., 2005). Notably, PGC-ablated fish develop either as sterile males, for example zebrafish (*Danio rerio*) (Slanchev et al., 2005), or as either sterile males or sterile females, for example loach (*Misgurnus anguillicaudatus*) (Fujimoto et al., 2010). The presence of a germline is required for phenotypic female sex determination in zebrafish (Siegfried and Nusslein-Volhard, 2008), but is not the primary determinant in goldfish (*Carassius auratus*) (Goto et al., 2012). Regardless of their role in sex determination, the presence of PGCs in the early gonad is a prerequisite for germline and gonadal development in teleosts. Therefore, basic knowledge of molecular events in PGCs is essential for understanding germline development. Molecular markers are powerful tools for identifying target cell types and stages of differentiation. In teleosts, germ cell marker genes exist for advanced germ cells, such as spermatogonia/oogonia, spermatocytes/oocytes, and spermatids, but also for PGCs (Xu et al., 2010).

Fish PGCs were first characterized in zebrafish using *vasa* as a germ cell marker gene (Olsen et al., 1997; Yoon et al., 1997). *Vasa*, a gene that codes for an ATP-dependent RNA helicase of the DEAD box protein family, is involved in RNA-dependent cellular processes (Linder and Lasko, 2006; Sengoku et al., 2006). Zygotic expression of *vasa* occurs strictly in the germline cells throughout life. Furthermore, its germ cell-specific expression pattern is highly conserved in a wide variety of organisms, from planaria to humans (Shibata et al., 1999; Castrillon et al., 2000). Notably, it was recently reported in medaka (*Oryzias latipes*) that *vasa* was not required for PGC proliferation and survival, but was still required for PGC migration (Li et al., 2009). The *dnd* gene encodes an RNA-binding protein that regulates germ cell viability and suppresses the formation of germ cell tumors, and is a component of germ plasm (also known as nuage) and germ cell granules inside vertebrate PGCs (Weidinger et al., 2003). Recent studies reported a novel function for Dnd1 in protecting certain mRNAs from miRNA-mediated repression. In zebrafish, Dnd1-deficient PGCs show a significant decrease in the expression of exogenously delivered *nos*1, *TDRD7* (Kedde et al., 2007), and *hub* mRNAs (Mickoleit et al., 2011), which have miR-430 seed sequences located in their 3′-UTRs. Interestingly, *lymphocyte antigen 75* (*ly75*) was recently identified as a mitotic germ cell-specific marker in rainbow trout (*Oncorhynchus mykiss*) by expressed sequence tag analyses derived from purified type A-spermatogonia cDNA library (Nagasawa et al., 2010). Information about Ly75 is limited to the immune system (East and Isacke, 2002), and its function has been known as an antigen-uptake receptor in dendritic cells (Jiang et al., 1995). Even though the role of Ly75 in germ cells remains to be uncovered, its expression in fish gonads is strictly limited to mitotic germ cells, including PGCs (Nagasawa et al., 2010). So far, PGC identification and their migratory pathway during embryogenesis have been investigated using germ cell marker genes in: Cyprinidae, including zebrafish (Yoon et al., 1997), goldfish (Otani et al., 2002), and rare minnow (*Gobiocypris rarus*) (Cao et al., 2012); Cobitidae, such as weather loach (Fujimoto et al., 2006); Adrianichthyidae, namely medaka (Herpin et al., 2007); Gobiidae, such as ukigori (*Gymnogobius urotaenia*) (Saito et al., 2004) and shiro-uo (*Leucopsarion petersii*) (Miyake et al., 2006); and Gadidae, namely Atlantic cod (*Gadus morhua*) (Presslauer et al., 2012).

Atlantic salmon (*Salmo salar*) is one of the most important aquaculture species worldwide, and has been the subject of intensive research due to its great commercial value. Most studies within salmon reproductive biology have been performed on spermatogenesis and/or oogenesis around puberty and sexual maturation since this process impairs fish growth and flesh quality (Celius and Walther, 1998; Maugars and Schmitz, 2008a, b). Nevertheless, knowledge of germline formation and development during early embryogenesis is crucial to develop efficient tools towards the control of fertility in the Atlantic salmon. A representative Salmonidae *vasa* was first cloned in rainbow trout (Yoshizaki et al., 2000a). Subsequent studies using *vasa-gfp* transgenic fish and chimeric RNA injection detected green fluorescence protein (GFP)-labeled PGCs in larvae of rainbow trout, masu salmon (*Oncorhynchu*s *masou*), brook trout (*Salvelinus fontinalis*), and brown trout (*Salmo trutta*) (Yoshizaki et al., 2000a, b, 2005; Sakao et al., 2009); no data currently exists for Atlantic salmon. Also, despite their biological and economic importance, no study has been reported yet on PGC identification and their migratory pathway during early embryogenesis in the superorder Protacanthopterygii in general, and in salmonids in particular. In this study, we aimed to identify an appropriate PGC marker gene in Atlantic salmon and to characterize PGC distribution during embryogenesis using whole-mount in situ hybridization and in vivo PGC labeling.

## RESULTS

### Characterization of Full-Length *vasa*, *dnd*, and *ly75* cDNAs in Atlantic Salmon

**Figure 1:**
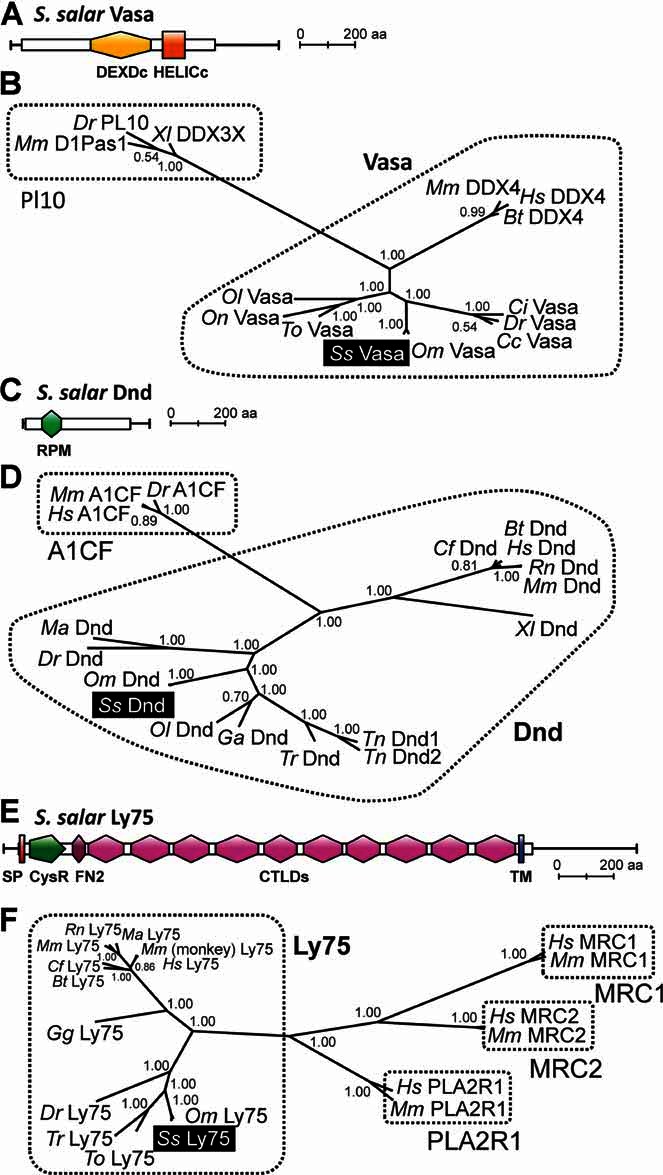
Protein domains and phylogenetic tree of Atlantic salmon *vasa*, *dnd*, and *ly75* genes. **A**: Protein domains of Atlantic salmon *vasa* amino acid sequences predicted by SMART. 5′- and 3′-UTRs (black lines) and coding region (white box) are indicated. DEAD-like helicases (DEXDc) and helicase superfamily c-terminal (HELICc) domains are shown. Scale bar shows 200 amino acids. **B**: Phylogenetic tree of *vasa* and PL10 found in vertebrates. Numbers at the nodes indicate posterior probability and approximate likelihood-ratio values obtained from the Bayesian method. Species abbreviations and their GenBank accession numbers are as follows: Vasa (*Bt*, *Bos Taurus*: NM_001007819; *Cc*, *Cyprinus carpio*: AF479820; *Ci*, *Ctenopharyngodon idella*: GQ140633; *Dr*, *Danio rerio*: NM_131057; *Hs*, *Homo sapiens*: NM_024415; *Mm*, *Mus musculus*: NM_010029; *Ol*, *Oryzias latipes*: AB063484; *Om*, *Oncorhynchus mykiss*: AB032566; *On*, *Oreochromis niloticus*: AB032467; *Ss*, *Salmo salar*: JN712912; *To*, *Thunnus orientalis*: EU253482) and PL10 (*Dr*: NM_130941;*Mm*: NM_033077; *Xl*, *Xenopus laevis*, NM_001086814). **C**: Protein domains of Atlantic salmon Dnd amino acid sequences predicted by SMART. 5′- and 3′-UTRs (black lines) and coding region (white box) are indicated. RRM domain is shown. Scale bar shows 200 amino acids. **D**: Phylogenetic tree of the Dnd and A1CF family found in vertebrates. Numbers at the nodes indicate posterior probability and approximate likelihood-ratio values obtained from the Bayesian method. Species abbreviations and their GenBank accession numbers are as follows: Dnd (*Bt*: NM_001007819; *Cf*, *Canis familiaris*: XM_843741; *Dr*: NM_212795; *Ga*, *Gasterosteus aculeatus*: ENSGACT00000025998 (Ensembl); *Hs*: NM_194249; *Ma*, *Misgurnus anguillicaudatus*: AB531494; *Mm*: NM_173383; *Ol*, NM_001164516; *Om*: NM_001124661; *Rn*, *Rattus norvegicus*: NM_001109379; *Ss*: JN712911; *Tn1*, *Tetraodon nigroviridis*: ENSTNIT00000007156 (Ensembl); *Tn2*: ENSTNIT00000000153 (Ensembl); *Tr*, *Takifugu rubripes*: ENSTRUT00000022988 (Ensembl); *Xl*: AY321494) and A1CF (*Dr*: XM_680086; *Hs*: *NM_014576*; *Mm*: NM_001081074). **E**: Protein domains of Atlantic salmon Ly75 amino acid sequences predicted by SMART. 5′- and 3′-UTRs (black lines) and coding region (white box) are indicated. SP, RICIN/CysR, FN2, CTLD, and TM domains are shown. Scale bar shows 200 amino acids. **F**: Phylogenetic tree of Ly75 and other members of the mannose receptor family found in vertebrates. Species abbreviations and their GenBank accession numbers are as follows: Ly75 (*Bt*: AY264845; *Cf*: XM_545488; *Dr*: XM_690165; *Gg*, *Gallus gallus*: AJ574899; *Hs*: AF011333; *Ma*, *Mesocricetus auratus:* AB059273; *Mm*, U19271; *Mm* (monkey), *Macaca mulatta*, XM_001093552; *Om*, GQ468309; *Rn*: XM_001068965; *Ss*: JN712913; *To*: GQ468310; *Tr*: AB438982), MRC1 (*Hs*: NM_002438; *Mm*, NM_008625), MRC2 (*Hs*, AF134838; *Mm*, NM_008626), and PLA2R1 (*Hs*, NM_008867; *Mm*, XM_039118). [Color figure can be viewed in the online issue, which is available at http://wileyonlinelibrary.com]

Atlantic salmon full-length *vasa* (JN712912) was 2,734 bp long and contained an open reading frame (ORF) of 1,962 bp, encoding 654 amino acids (Fig. S1A). Multiple sequence alignments showed that salmon Vasa was 94% and 79% identical to Vasa of rainbow trout and zebrafish, respectively. Domain structure analysis using SMART revealed DEAD-like helicases (DEXDc) at amino acid positions 236–447 and helicase super family C-terminal (HELICc) domains at positions 483–564 (Fig. 1A). Phylogenetic analysis using the Bayesian inference method showed that salmon Vasa clustered with other teleost Vasa protein sequences, and was closely related to rainbow trout Vasa (Fig. 1B).

The full-length Atlantic salmon *dnd* (JN712911) was 1,326 bp long and contained an ORF of 1,101 bp, which encoded 367 amino acids (Fig. S1B). Multiple alignments showed that salmon Dnd shared 96% and 47% identity with Dnd of rainbow trout and zebrafish, respectively. SMART revealed an RNA recognition motif (RRM) at amino acid positions 54–127 (Fig. 1C). The Bayesian phylogenetic analysis of Dnd protein with a related protein, A1CF, revealed that teleost Dnd formed a distinct cluster from tetrapods and amphibian Dnd proteins, and salmon Dnd showed a close association to rainbow trout Dnd (Fig. 1D).

The full-length Atlantic salmon *ly75* (JN712913) was 6,526 bp long, containing an ORF of 5,307 bp that encoded 1,769 amino acids (Fig. S1C). Multiple alignments showed that salmon Ly75 had identities of 93% and 50% to Ly75 of rainbow trout and zebrafish, respectively. SMART revealed several conserved domains, namely a signal peptide (SP) at amino acid residues 1–21; a RICIN-type beta-trefoil (RICIN/CysR) at positions 33–161; fibronectin type 2 (FN2) at positions 180–228; C-type lectin domains (CTLD) at positions 235–361, 382–516, 529–653, 672–823, 841–957, 978–1,115, 1,126–1,243, 1,260–1,403, 1,415–1,545,and 1,567–1,708; and a transmembrane (TM) domain at positions 1,717–1,739 (Fig. 1E). The Bayesian phylogenetic reconstruction clearly separated the members comprising the mannose receptor family (Ly75, MRC1, MRC2, and PLA2R1) to four respective clusters according to protein subfamily (Fig. 1F). Both teleost and tetrapods Ly75 were grouped to each clade in a Ly75 cluster according to the generally accepted species relationship. Salmon Ly75 was closely related to rainbow trout Ly75.

### Tissue Distribution of *vasa*, *dnd*, and *ly75* Transcripts in Adult Fish

**Figure 2:**
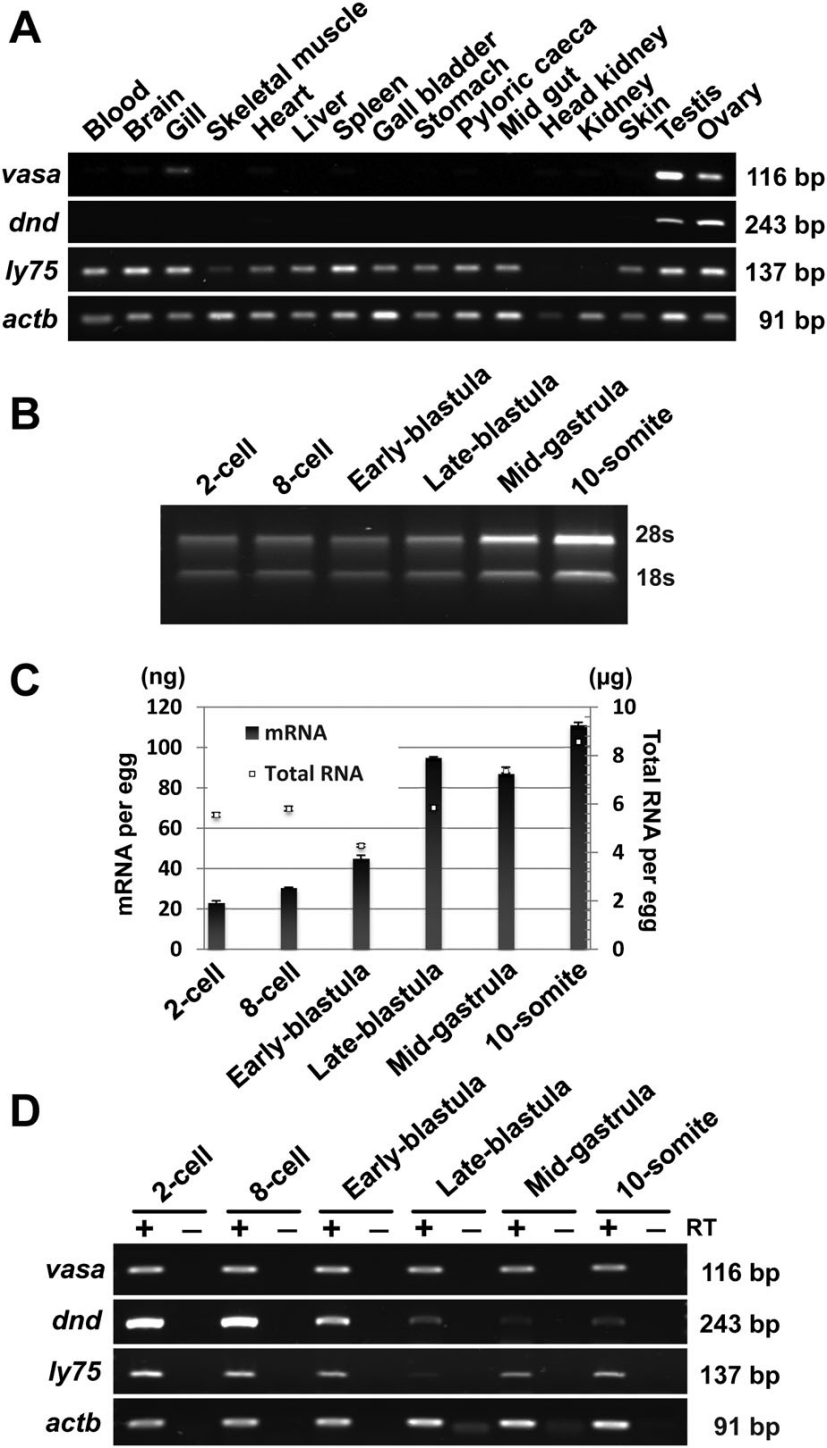
Distributionof *vasa*, *dnd*, and *ly75* transcripts in Atlantic salmon. **A**: cDNA from various tissues of adult fish (blood, brain, gill, skeletal muscle, heart, liver, spleen, gall bladder, stomach, pyloric caeca, mid gut, head kidney, kidney, skin, testis, and ovary) were used for semi-quantitative RT-PCR. *Actb* was used as endogenous reference. Amplicon sizes, in base pairs, are indicated on the right. Expression pattern was determined using two biological replicates. **B**: Total RNA (400–900 ng) from early embryonic stages (two-cell, eight-cell, early-blastula, late-blastula, mid-gastrula, and 10-somite) was electrophoresed. Both 28S and 18S rRNA, stained with SYBR Safe DNA gel stain, are shown in all stages. **C**: The changes of both total RNA (white squares) and mRNA (black bars) amount per egg for each developmental stage. The concentration was quantified using three replicates. **D**: cDNA synthesized from above-mentioned developmental stages were used for semi-quantitative RT-PCR. In order to eliminate a possibility of genomic DNA contamination, −RT (without reverse transcriptase) samples of each counterpart were examined and electrophoresed. Amplicon sizes, in base pairs are indicated on the right.

*vasa* and *dnd* mRNAs were specifically detected in Atlantic salmon testes and ovaries. No expression was detected in other tissues, although we observed weak detection of *vasa* mRNA in gills. *Ly75* mRNA was expressed in most tissues, including gonads, with the exception of the pronephros and excretory kidney (Fig. 2A).

### Detection of *vasa*, *dnd*, and *ly75* Transcripts During Early Embryogenesis

Total RNA was derived from different stages (two-cell to 10-somite stages), assessed for their integrity by electrophoresis, and used for mRNA purification. Both 28S and 18S ribosomal RNA fragments were clearly observed (Fig. 2B), indicating the high quality of extracted RNA. The proportion of mRNA in the total RNA isolated from different stages of embryogenesis dramatically increased during blastulation, from 45 to 95 µg per egg, even though the total RNA content was equal (4.3–5.8 µg, Fig. 2C). Reverse-transcriptase-PCR (RT-PCR) analyses showed that *vasa*, *dnd*, and *ly75* mRNA were present at the two-cell stage (Fig. 2D). *vasa* mRNA was consistently detected with a relatively high-expression level throughout embryogenesis (two-cell to 10-somite stages). *dnd* mRNA, on the other hand, was highest at the two-cell stage, followed by a gradual decrease during cleavages and blastulation, but remained detectable at a reduced level during somitogenesis (Fig. 2D). While maternal ly75 mRNA was also gradually degraded by late-blastula stage, embryonic *ly75* mRNA was first detected from mid-gastrula stage onwards (Fig. 2D).

### Localization of *vasa*, *dnd* Transcripts, and ly75 Protein to the Genital Ridges of Larvae

**Figure 3:**
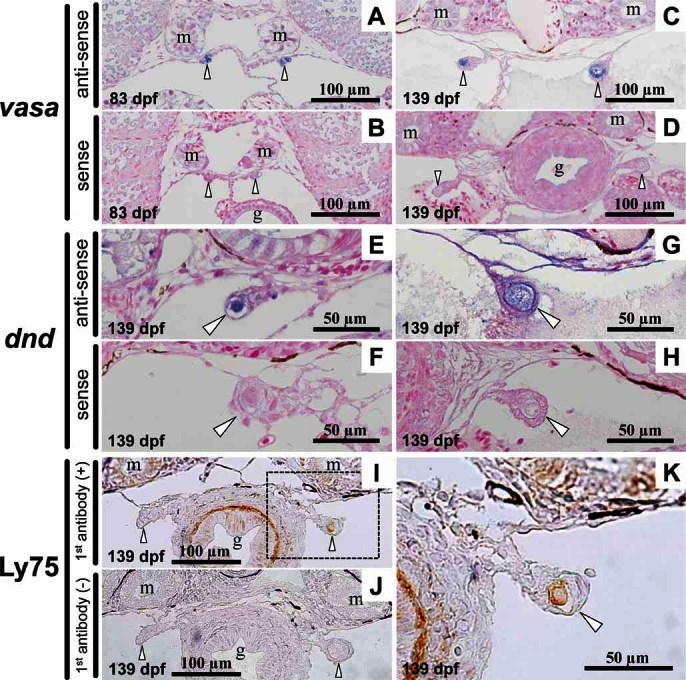
Localization of *vasa* and *dnd* transcripts or Ly75 protein in the genital ridge of Atlantic salmon larva. In situ hybridization with *vasa* (anti-sense: **A** and **C**; sense: **B** and **D**) or *dnd* (anti-sense: **E** and **G**; sense: **F** and **H**) probes, and immunohistochemistry with Ly75 antibody (**I** and **K**) or without primary antibody (**J**). (**A**, **B**) and (**C–K**) are hatching (83 dpf) and yolk-sac resorption stages (139 dpf), respectively. (**K**) is a high-magnification view of genital ridge area enclosed by dashed box in (**I**). Embryos were fixated with PFA (A, B, E, F, and I–K) or Bouin's solution (C, D, G, and H). Arrowheads indicate the genital ridges. g, gut; m, mesonephric duct. [Color figure can be viewed in the online issue, which is available at http://wileyonlinelibrary.com]

Both *vasa* and *dnd* mRNAs were specifically detected in PGCs of Atlantic salmon in larvae sections (Fig. 3A–H). At hatching (83 days post-fertilization; dpf), *vasa* mRNA could be found in PGCs that were symmetrically distributed in bilateral positions at the presumptive region of genital ridges (Fig. 3A). At the yolk-sac resorption stage (139 dpf), *vasa*-expressing PGCs were surrounded by gonadal somatic cells within the forming genital ridges, which are located peripherally along the wall of abdominal cavity (Fig. 3C). At the same stage (139 dpf), *dnd* mRNA was observed in PGCs, although expressed at a lower level than *vasa* (Fig. 3E,G). No signal was observed in the hybridization with sense probes of *vasa* (Fig. 3B,D) or *dnd* (Fig. 3F,H). In situ immunodetection revealed germ cell-specific localization of Ly75 protein in PGCs within the genital ridge (139 dpf, Fig. 3I,K), whereas no signal was detected in the control samples (without the primary antibody, Fig. 3J).

### Identification of Salmon PGCs During Early Embryogenesis

**Figure 4:**
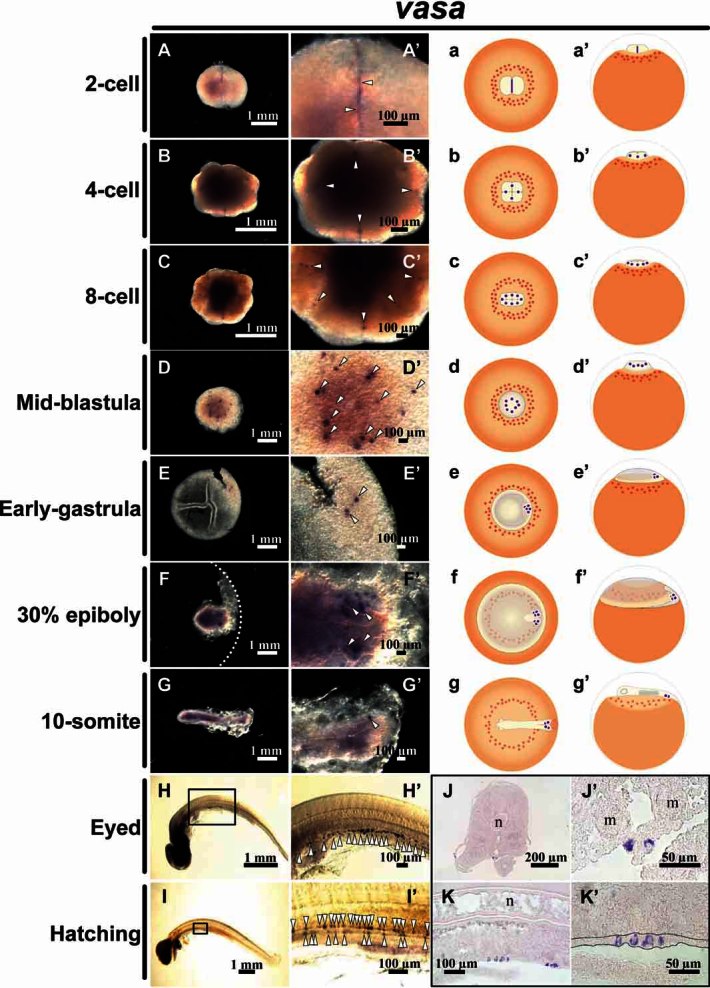
Localization patterns of *vasa* transcripts and PGCs in Atlantic salmon embryo. Whole-mount in situ hybridization with *vasa* probe at different developmental stages (**A–I**, **A**′**–I**′). Transverse or longitudinal sections of the embryo subjected to hybridization with the *vasa* probe at the beginning of eye-pigmented stage (41 dpf) (**J** and **K**, **J**′ and **K**′). (**A**′–**K**′) are high-magnification views of (**A–K**), respectively. Arrowheads indicate the localization of *vasa* transcripts. Dotted lines in **F** and **K**′ indicate the edges of blastoderm and presumptive genital ridge, respectively. m, mesonephric duct; n, notochord (**a**–**g**, **a**′–**g**′). The schematic representation of the localization *vasa* transcripts and PGC distribution in the Atlantic salmon embryo at two-, four-, eight-cell, mid-blastula, early-gastrula, 30% epiboly, and 10-somite stages. The *vasa* signals and PGCs are represented in purple (line or dots) in the schematic representation. (**a**–**g**) and (**a**′–**g**′) are anterior and lateral views of embryo, respectively.

No obvious *vasa* mRNA signal was observed in the Atlantic salmon blastodisc at the one-cell stage, indicating that *vasa* mRNA was broadly distributed throughout the blastodisc at levels undetectable by in situ hybridization (data not shown). Instead, *vasa* mRNA was first clearly detected in the cleavage plane at the two-cell stage (1 dpf, Fig. 4A,A′). At the four-cell stage (1.5 dpf), *vasa* mRNA was aggregated in four spots localized at both ends of the first and second cleavage planes (Fig. 4B,B′). Eight spots of *vasa* mRNA were subsequently detected at the both ends of all cleavage planes at the eight-cell stage (2 dpf, Fig. 4C,C′). At mid-blastula, several spots of *vasa* mRNA could be observed in the central region of the blastodisc (7 dpf, Fig. 4D,D′). At the start of epiboly (13 dpf), *vasa* mRNA was seen at the presumptive region of the embryonic shield in the blastoderm (Fig. 4E,E′). During early-gastrulation (17 dpf), clusters of *vasa* transcripts were symmetrically distributed on both sides of the embryonic shield (Fig. 4F,F′). During somitogenesis (27–51 dpf), *vasa* mRNA signal gradually distributed along the developing gonadal region from the posterior to anterior side (Fig. 4G,G′ and H,H′), and then formed bilateral lines corresponding to the genital ridges at hatching (83 dpf, Fig. 4I,I′). At the beginning of the pigmented eye stage (41 dpf), the cells expressing *vasa* could be first seen in the presumptive genital ridge, below the mesonephric ducts (Fig. 4J,J′ and K,K′). Very little staining was observed with the *vasa* sense probe at any developmental stage examined. The number of the cells expressing *vasa* was 33.0 ± 1.7 (mean ± standard deviation, n = 25) at 41 dpf and 53.5 ± 4.0 (n = 11) at hatching (83 dpf).

### Visualization of Salmon PGCs by Microinjection of *gfp-rt-vasa* 3′-UTR RNA

**Figure 5:**
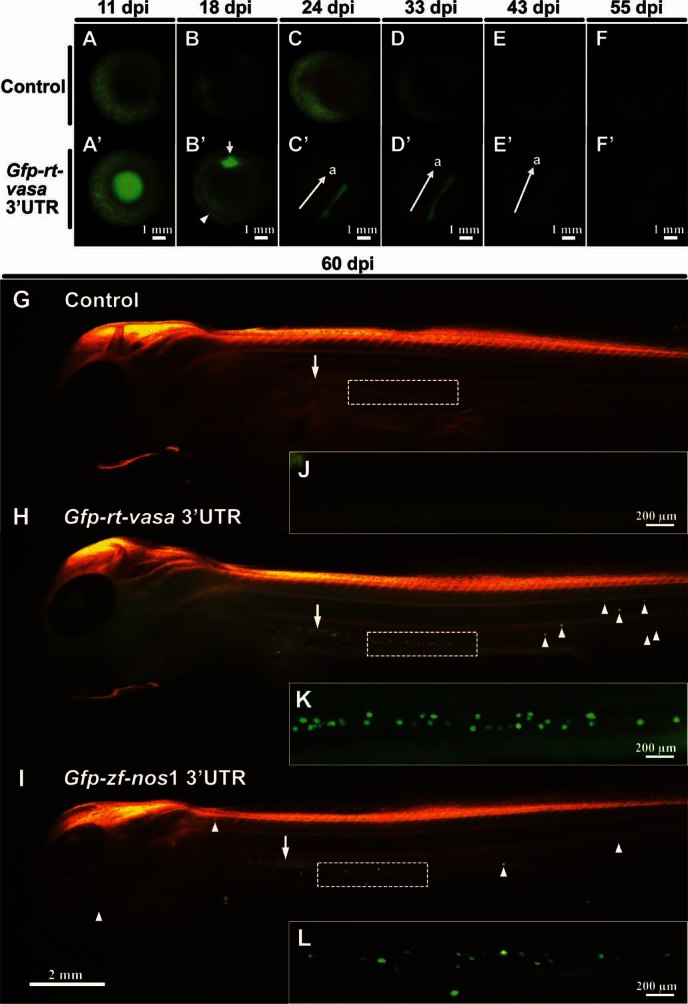
Sequential tracking of GFP translated from *gfp-rt-vasa* 3′-UTR RNA in Atlantic salmon embryo. The fluorescent views of the control embryo (non-injected embryo; **A**–**F** and **G**, **J**), the *gfp-rt-vasa* 3′-UTR RNA**-**injected embryo (**A**′–**F**′ and **H**, **K**), or the *gfp-zf-nos*1 3′-UTR RNA**-**injected embryo (**I**, **L**). Sequential GFP localization is observed in the *gfp-rt-vasa* 3′-UTR RNA**-**injected embryo throughout embryogenesis as follows: **A**′: Blastodisc (11 days post-injection, dpi) showing ubiquitous GFP expression at late-blastula stage. **B**′: GFP expression in the embryonic shield as a thickened margin (arrow) and at the edge of blastoderm (arrowhead) along with epiboly movement at pre-mid-gastrula stage (30% epiboly, 18 dpi). **C**′–**F**′: Declining GFP in the somatic cells of embryo during somitogenesis (24–55 dpi). In some cases, the yolk shows auto fluorescence. Arrows in **C**′–**E**′ show the direction of body axis a, anterior. **G**–**I**: Lateral view of head to trunk region of embryos at 60 dpi under fluorescence. **J**–**L**: High-magnification views of the genital ridge area, indicated by dashed boxes (**G**–**I)**. Arrows and arrowheads indicate weak auto fluorescence in the mesonephric duct, and the positions where the GFP-expressing cells were observed on the outside of genital ridge area, respectively.

In eggs injected with *gfp-rt-vasa* 3′-UTR RNA, the fluorescence signal could be observed first at the whole area of blastodisc at the late-blastula stage, about 11 days post-injection (dpi) (Fig. 5A′). From the onset of gastrulation, the fluorescence signal in the blastoderm diminished. Relatively strong signal was observed in the embryonic shield and in the thickened edge of blastoderm at the pre-mid-gastrula stage (18 dpi, Fig. 5B′). A gradual decrease in fluorescence signal was observed in the embryonic body during somitogenesis (24–55 dpi) (Fig. 5C′–F′). At 60 dpi, GFP-positive cells could be detected in the genital ridge region attached to the abdominal wall (Fig. 5H,K). No GFP expression was found in control embryos (non-injected embryos) throughout embryogenesis (Fig. 5A–G, and J). In addition, GFP-positive cells could also be found in the genital ridge regions of embryos injected with *gfp-zf-nos*1 3′-UTR RNA (60 dpi, Fig. 5I,L). Weak fluorescence was observed in whole body of the embryos injected with *gfp-rt-vasa* 3′-UTR RNA (60 dpi, Fig. 5H), but not in the controls (60 dpi, Fig. 5I) or the embryos injected with *gfp-zf-nos*1 3′-UTR RNA (60 dpi, Fig. 5I). PGCs labeled with *gfp-rt-vasa* 3′-UTR RNA showed stable and high fluorescence intensity (Fig. 5K), whereas PGCs labeled with *gfp-zf-nos*1 3′-UTR RNA displayed variable GFP expression at low fluorescence intensities (Fig. 5L). A few ectopic GFP-positive cells were present in the head and tail regions of embryos injected with both constructs (60 dpi, Fig. 5H,I).

## DISCUSSION

In the present study, we cloned the full-length Atlantic salmon *vasa*, *dnd*, and *ly75* cDNAs, and characterized their expression patterns during embryogenesis. In semi-quantitative RT-PCR analyses, we revealed that salmon *vasa* and *dnd* genes were specifically expressed in both male and female gonads, as reported in other teleosts (Olsen et al., 1997; Yoon et al., 1997; Yoshizaki et al., 2000a; Otani et al., 2002; Weidinger et al., 2003; Liu et al., 2009; Nagasawa et al., 2009; Raghuveer and Senthilkumaran, 2010; Blazquez et al., 2011; Cao et al., 2012; Lin et al., 2012b; Presslauer et al., 2012). Salmon *ly75* transcripts were expressed in several tissues and were particularly abundant in testis and ovary; a similar distribution pattern was observed in rainbow trout and bluefin tuna *ly75* (Nagasawa et al., 2010, 2012b). During embryogenesis, *vasa*, *dnd*, and *ly75* transcripts were already present at the two-cell stage, with the highest mRNA levels throughout early embryogenesis between the two-cell and 10-somite stages. This suggested that the above three transcripts are maternally inherited, similarly to other teleost *vasa* and *dnd* homologs (Olsen et al., 1997; Yoon et al., 1997; Weidinger et al., 2003). Previously, while rainbow trout *ly75* mRNA was predominantly detected in oogonia and chromatin nucleolus-stage oocytes in the ovary, an extremely weak *ly75* mRNA signal was partially observed in more advanced oocytes (Nagasawa et al., 2010). Therefore, this molecule had been considered a non-maternal component. The current report clearly shows that the presence of maternally deposited *ly75* transcripts in the eggs of Atlantic salmon. Interestingly, after the disappearance of maternal *ly75* transcripts at the late-blastula stage (Fig. 2D), an increase in *ly75* transcripts was observed from mid-gastrula stage. This was likely as a result of zygotic gene expression at the mid-blastula stage, as reported in the closely related species, rainbow trout (Takeuchi et al., 1999). It should be noted that the gradual decrease in maternally deposited *dnd* transcripts was also observed during embryonic development in zebrafish and medaka (Weidinger et al., 2003; Liu et al., 2009).

In situ hybridization and immunohistochemistry confirmed the expression of the three germ cell marker candidates in PGCs in the genital ridges at hatching (83 dpf) and yolk-sac resorption stages (139 dpf, Fig. 3). *vasa* and *dnd* mRNAs were found in PGCs, and were constantly detected at high (*vasa*) and low (*dnd*) levels during larval stages. Atlantic salmon PGCs are 20–25 µm in diameter, similar to rainbow trout PGCs (Okutsu et al., 2006; Nagasawa et al., 2010). Immunostaining with an antibody against rainbow trout Ly75 showed that within the genital ridge, salmon Ly75 protein specifically localized in PGCs. This germ cell-specific expression of gonadal Ly75 seems to be highly conserved in all the fish species studied to date (Nagasawa et al., 2010, 2012b). Eventually, we concluded that amongst *dnd*, *ly75*, and *vasa*, the latter was the most appropriate marker gene for identifying PGCs by whole-mount in situ hybridization throughout salmon embryogenesis from the standpoint of its transcription level, expression pattern, and specificity in germ cells.

Salmon *vasa*, a putative germ plasm component, exhibited a distribution pattern slightly different to *vasa* homologs in Cyprinidae and Gobiidae by whole-mount in situ hybridization (Yoon et al., 1997; Koprunner et al., 2001; Weidinger et al., 2003). Specifically, the typical *vasa* localization pattern showing four clusters during cleavage and early-blastula stages (Raz, 2002) was not observed from the eight-cell stage. Furthermore, at the mid-blastula stage, *vasa*-expressing cells were randomly distributed at the central part of blastodisc (7 dpf, Fig. 4D). Therefore, specific differences in distribution of Atlantic salmon *vasa* mRNA and *vasa*-expressing cells have been observed during cleavage and blastulation. The distribution pattern of *vasa*-expressing PGCs between 30% epiboly to hatching stages, examined in this study, seems to be highly conserved amongst distant phyla, such as in the Cypriniformes (e.g., zebrafish and rare minnow) (Raz, 2002; Cao et al., 2012), representing the superorder Ostariophysii, as well as in Gadiformes (e.g., Atlantic cod) (Presslauer et al., 2012), representing superorder Paracanthopterygii, and in Pleuronectiformes (e.g., turbot, *Scophthalmus maximus*) (Lin et al., 2012b), representing superorder Acanthopterygii. The current study is the first report on *vasa* mRNA distribution during embryogenesis in a representative of another superorder, Protacanthopterygii. It is noteworthy that the cells expressing *vasa* in the presumptive genital ridge of Atlantic salmon were quantifiable from the beginning of the eye-pigmented stage (41 dpf). Also, their number was relatively lower than the rainbow trout PGC counts at same stage (Yoshizaki et al., 2000a; Nagler et al., 2011).

As an alternative approach for identifying PGCs, we visualized salmon PGCs in vivo by injecting chimeric RNA comprised of two sequences, the coding region of *gfp* and 3′-UTR of *vasa* (Koprunner et al., 2001; Yoshizaki et al., 2005; Kurokawa et al., 2006; Saito et al., 2006, 2011; Lin et al., 2012a) or *nos*1 (Saito et al., 2006, 2011; Lin et al., 2012a). The rainbow trout *vasa* 3′-UTR has been previously shown to play a critical role in stabilizing mRNA in PGCs of several Salmonidae species (Yoshizaki et al., 2005), while the zebrafish *nos*1 3′-UTR has been reported to be stabilized in PGCs of various fish species, such as eel (Saito et al., 2011) or loach (Saito et al., 2006). As the first step in visualizing salmon PGCs in vivo, the above two xenogeneic 3′-UTR sequences were chosen and used to obtain GFP expression in Atlantic salmon PGCs instead of endogenous salmon *vasa* or *nanos* genes; however, further studies would be required to confirm their mRNA stability in this species. In the present report, the GFP signal intensity from *gfp-rt-vasa* 3′-UTR RNA gradually decreased in somatic cells after blastula stage because of the degradation of injected chimeric RNA. In contrast, GFP was constantly detected in PGCs, indicating that injected chimeric RNA was specifically stabilized in PGCs throughout embryogenesis. A similar pattern of PGC-specific mRNA stabilization was observed in the embryos injected with *gfp-zf*-*nos*1 3′-UTR RNA. These data clearly support the hypothesis that xenogeneic 3′-UTR sequences of *vasa* and *nos*1 retain their functions in salmon PGCs, and the transcripts they are associated with are specifically protected against common RNA degradation mechanisms, such as miRNA-mediated processing (Kedde et al., 2007). Remarkably, GFP-labeled salmon PGCs displayed sufficient green fluorescence intensity in genital ridges for at least 87 dpi. This technique enables in vivo identification and isolation of viable PGC by fluorescent activated cell sorting (Kobayashi et al., 2004). Furthermore, the isolated PGCs have potential use for further studies, such as transplantation, cell culture (Okutsu et al., 2006; Shikina and Yoshizaki, 2010), and molecular analyses using next-generation sequencing technologies.

In conclusion, the present study demonstrated the evaluation of potential germ cell markers and their expression in early developmental stages of Atlantic salmon. This is the first report amongst the superorder Protacanthopterygii. Whole-mount in situ hybridization analysis of *vasa* mRNA revealed that salmon PGC specification and migration during cleavage and blastula stages had a unique pattern from that of other fish species studied so far. These findings are the first step to understand germline specification in Atlantic salmon, along with its applications in reproductive biotechnology, such as induced sterility through targeted cell ablation or PGC manipulation.

## MATERIALS AND METHODS

### Sample Collection

Two-year-old Atlantic salmon were maintained in land-based tanks in research facility at Mørkvedbukta Research Station (University of Nordland, Bodø, Norway). Nine fish of 38.9 ± 2.2 cm fork length and 635.1 ± 107.2 g body weight (mean ± standard deviation) were humanely killed by immersion in seawater containing 1 g · L^−1^ tricaine methane sulfonate (Sigma–Aldrich, Oslo, Norway). The various organs or tissues (blood, brain, gill, skeletal muscle, heart, liver, spleen, gall bladder, stomach, pyloric caeca, mid gut, head kidney, kidney, skin, testis, and ovary) were excised, snap-frozen in liquid nitrogen, and stored at −80°C until RNA extraction. Gonadosomatic index (100 × gonad weight/total body weight) was 0.07 ± 0.05% for males (mean ± standard deviation, n = 5) and 0.14 ± 0.03% for females (n = 4). Unfertilized eggs and sperm from three females and three males were generously provided by AquaGen AS (Trondheim, Norway). Upon collection, gametes were processed as described by Babiak and Dabrowski (2003), transported overnight on crushed ice, then fertilized according to the general protocol (Gorodilov, 1996). The fertilized eggs were transferred to plastic containers filled with freshwater and reared in refrigerated cell incubators (Sanyo, Watford, UK) at 6°C over 3 months. Approximately 50 eggs of each developmental stage (Table 1) were snap-frozen for RNA extraction and sampled for in situ hybridization analyses. All procedures were conducted in accordance to the guidelines set by the National Animal Research Authority (Forsøksdyrutvalget, Norway).

**TABLE 1:** Overview of Developmental Stages, Incubation Time, and Accumulated Temperature (°C × days) of Atlantic Salmon Embryos and Larvae Sampled

Sub period	Developmental stage	Time	Accumulated temperature
Fertilization	Unfertilized	—	—
Cleavage	1-cell	8 hpf	2
	2-cell	28 hpf	7
	4-cell	35 hpf	9
	8-cell	48 hpf	12
	16-cell	51 hpf	13
	32-cell	56 hpf	14
	64-cell	63 hpf	16
	128-cell	69 hpf	17
Blastulation	Early-blastula	5 dpf	30
	Mid-blastula	7 dpf	42
	Late-blastula	10 dpf	60
Gastrulation	Early-gastrula (10% epiboly)	13 dpf	78
	Pre-mid-gastrula (30% epiboly)	17 dpf	102
	Mid-gastrula (50% epiboly)	21 dpf	126
	Late-gastrula (90% epiboly)	24 dpf	144
Somitogenesis	10-Somite	27 dpf	162
	eyed (65-somite)	51 dpf	306
Larva	Hatching	83 dpf	498
	yolk-sac resorption	139 dpf	834

Incubation time is represented by hour post-fertilization (hpf) or day post-fertilization (dpf).

### Cloning Full-Length cDNA Sequences of *vasa*, *dnd*, and *ly75* Genes in Atlantic Salmon

The composition of cloned cDNA regions covering the full-length *vasa*, *dnd*, and *ly75* cDNA sequences in Atlantic salmon are detailed in Table 2. Total RNA was extracted from both testis and ovary, and used for cDNA synthesis as previously reported (Campos et al., 2010). Internal regions of *vasa*, *dnd*, and *ly75* cDNAs were amplified by PCR with gene-specific and/or degenerate primers that were designed against the conserved regions across fish orthologs (Table 2). Subsequently, 5′- and 3′-end regions of above cDNAs were amplified by 5′- and 3′-rapid amplification of cDNA ends using a GeneRacer kit (Life Technologies, Paisley, UK) with gene-specific primers (Table 2) according to the manufacturer's instructions. Amplified PCR fragments were cloned and sequenced as described elsewhere (Campos et al., 2010).

**TABLE 2:** Fragment Regions, Primer Sequences, Amplicon Sizes (bp), and GenBank Accession Numbers of Atlantic Salmon *vasa*, *dnd*, *ly75*, and *actb* Genes Amplified in the Study

Gene	Type of PCR	Region		Sequence (5′–3′)	Size	GenBank
*vasa*	5′RACE	1–1,154	Fw:	CGACTGGAGCACGAGGACACTGA	1,154	JN712912
			Rv:	TGCAGCCCTTCAGTATCTCACGAATGGT		
	PCR	979–2,083	Fw:	TCAGTTCAGCGAGATCCAGGAGCCAGA	1,105	
			Rv:	TCATCACTCCCATTCGTCGTCGTCT		
	3′-RACE	1,965–2,734	Fw:	TGTGGGAGAACCTTCGCCTCCACTGATAG	770	
			Rv:	GCTGTCAACGATACGCTACGTAACG		
	RT-PCR	1,513–1,628	Fw:	GACTACAGGGTCTGAACGCA	116	
			Rv:	CGCGGTCACCATGAATACTA		
*dnd*	5′-RACE	1–290	Fw:	GGACACTGACATGGACTGAAGGAGTA	290	JN712911
			Rv:	TCATCATGAGGCGGAACTCCCAGAGAGG		
	PCR	116–504	Fw:	ACYCARGTYAAYGGSCAGAGRAARTATGG	389	
			Rv:	TCAGAGAAGTCCAGCAGCACCTGCAGCAG		
	3′-RACE	310–1,326	Fw:	TGGCTTTGCCTACGCCAAGTACGACAGC	1,017	
			Rv:	CGCTACGTAACGGCATGACAGTG		
	RT-PCR	18–260	Fw:	CGAGACCTAGGATAATGGAGGAGCGT	243	
			Rv:	CCACGGCACGGAACAGCGGAATCAG		
*ly75*	5′-RACE	1–648	Fw:	CGACTGGAGCACGAGGACACTGA	648	JN712913
			Rv:	TCGGTCGACTCATCCCTCCTCCAGGAGT		
	PCR	419–1,970	Fw:	TCCGGCCACCGTCTCTTCCACGT	1,552	
			Rv:	CCGAGCCATCCTGAGTGACCCACTGGTA		
	PCR	1857–4,107	Fw:	TCATCAATAGACTCCTTGCAGAAGAGAT	2,251	
			Rv:	TAACTCATTCTCCGCTAAGTTCCTGAT		
	PCR	3934–5,218	Fw:	TCCTCACAAGAGCGGCGGACCAAACT	1,285	
			Rv:	TGCAGACACCATGACAGCACAGGAGT		
	RT-PCR	4,865–4,987	Fw:	AGTGGCTCGTCTAAGTGGGT	123	
			Rv:	CTGTGCATCAAGCCTTTCAC		
*actb*	RT-PCR	—	Fw:	CCAAAGCCAACAGGGAGAAG	91	BG933897
			Rv:	AGGGACAACACTGCCTGGAT		

### Bioinformatic Analyses

Deduced amino acid sequences of *vasa*, *dnd*, and *ly75* genes were obtained from complete coding sequences by using EMBOSS Transeq (http://www.ebi.ac.uk/Tools/st/emboss_transeq/). Sequence similarities were analyzed by blastp algorithm (blast.ncbi.nlm.nih.gov). Domain structure analysis was carried out with SMART (Simple modular Architecture Research Tool; smart.embl-heidelberg.de/) with the normal mode. Amino acid sequences were aligned with the corresponding orthologs in various species using MUSCLE (drive5.com). The resulting multiple sequence alignments was used for Bayesian phylogenetic analysis (MrBayes v3.1.2, mrbayes.csit.fsu.edu) as detailed elsewhere (Nagasawa et al., 2012a). Bayesian phylogenetic trees were obtained from a mixed model of amino acid substitution (1,000,000 generations, sampling every 10^th^ generation and burning at the first 10,000 trees). Graphical representations of phylogenetic trees were obtained with PhyloWidget (phylowidget.org).

### Semi-Quantitative RT-PCR

cDNA from adult fish was synthesized from total RNA (1 µg) extracted from the organs mentioned above by using the QuantiTect reverse transcription kit (Qiagen, Nydalen, Sweden). cDNA from embryonic stages was transcribed with above-mentioned kit from mRNA (60 ng) purified from the total RNA pool derived from 10 whole-egg homogenate of each developmental stage (two-, eight-cell, early-blastula, late-blastula, mid-gastrula, and 10-somite), as detailed in Table 1. Total RNA and purified mRNA were electrophoresed on a 1% (w/v) agarose gel to assessed RNA integrity, and were further quantified with a NanoDrop ND-1000 (Thermo Scientific, Saven & Werner AS, Kristiansand, Norway). Since there were some difficulties in RNA extraction from salmonid egg because of huge yolk mass that may contain compounds inhibiting cDNA synthesis or PCR, mRNA purification was carried out using a Dynabeads mRNA purification kit for mRNA purification from total RNA preps (Life Technologies) prior to cDNA synthesis. PCR reactions were conducted with recombinant Taq DNA Polymerase (Life Technologies), using primer sets detailed in Table 2. In order to eliminate the possibility of contamination with genomic DNA, −RT samples (without reverse transcriptase in cDNA synthesis) for each developmental stage was concurrently examined. Thermocycling parameters were 94°C for 3 min, followed by 35 cycles for *vasa* or 45 cycles for *dnd* and *ly75* or 25 cycles for *actb* of 30 sec at 94°C, 30 sec at 58°C (62°C for *dnd*), and 30 sec at 72°C, with a final elongation step of 72°C for 3 min. PCR products were analyzed by electrophoresis on a 1.2% (w/v) agarose gel, then visualized and photographed on a Kodak gel documentation system v.4.0.5 (Oslo, Norway).

### In Situ Hybridization

Digoxigenin-labeled sense and anti-sense RNA probes were individually synthesized from corresponding regions: *vasa*, nucleotides 1,965–2,734 (1,105 bps); *dnd*, nucleotides 310–1,326 (1,017 bps) (Table 2), as detailed elsewhere (Fernandes et al., 2006). For fixation, the chorion of an egg was punctured using fine forceps (DUMONT #55 forceps, Fine Science Tools, Heidelberg, Germany), and the whole egg was fixated with 4% paraformaldehyde (PFA)/PBS or Bouin's solution at 4°C for 12–24 hr. After washing out the fixative, the blastodisc, blastoderm, or embryo, depending on developmental stage, were mechanically excised from the yolk part. Whole-mount in situ hybridization was performed with PFA-fixed embryos, as reported by Fernandes et al. (2008). To reduce background signal, destaining with 100% EtOH was performed, and then embryos were mounted in 50% glycerol. Embryos were observed under a binocular microscope (Stemi SV11, Carl Zeiss, Oslo, Norway). For histological observations of embryos subjected to whole-mount in situ hybridization with the *vasa* probe, specimens (the beginning of eye-pigmented stage, 41 dpf) were dehydrated with ethanol series and embedded in paraffin. Sections of 4-µm thickness were mounted on glass slides, and then counter-stained with Eosin-Y (Microm International, Walldorf, Germany). Meanwhile, the in situ hybridization with paraffin sections of PFA- or Bouin's solution-fixed specimens (hatching stage, 83 dpf and yolk-sac resorption stage, 139 dpf) was performed as described previously (Nagasawa et al., 2009). Mounted sections were observed under a BX-51 microscope (Olympus, Oslo, Norway) and photographed with a scale. The schematic representation of salmon embryo development and PGC distribution were illustrated using Adobe Illustrator CS4 (Adobe Systems, Tokyo, Japan).

### Immunohistochemistry

Paraffin sections of PFA-fixed individual (yolk-sac resorption stage, 139 dpf) were treated with HistoVT One solution (Nacalai Tesque Inc., Kyoto, Japan) at 90°C for 20 min for antigen retrieval. Pre-absorbed primary antisera against rainbow trout Ly75 (recognition site; amino acids 238–509, according to GQ468309) prepared in a previous study (Nagasawa et al., 2010) cross-reacted to Atlantic salmon Ly75 antigen. The amino acid sequence identity of the antibody recognition site between rainbow trout Ly75 and Atlantic salmon Ly75 (amino acid residues 235–516, according to JN712913) showed 89% similarity and 88% identity. The immunostaining was carried out as detailed elsewhere (Nagasawa et al., 2010).

### *gfp-rt-vasa* 3′-UTR RNA Microinjection and Observations

*gfp-rt-vasa* 3′-UTR RNA (*gfp*-coding sequences fused with rainbow trout *vasa* 3′ UTR sequences) was synthesized by in vitro transcription using mMESSAGE mMACHINE T7 kit (Life Technologies, Paisley, UK), as described previously (Yoshizaki et al., 2005). The *gfp-zf-nos*1 3′-UTR RNA (*gfp*-coding sequences fused with zebrafish *nos*1 3′-UTR sequences) was synthesized from a construct, as detailed elsewhere (Saito et al., 2011). Synthesized transcripts were dissolved in diethylpyrocarbonate (DEPC)-treated water at a final concentration of 400 ng/µl. The microinjection of *gfp-rt-vasa* or *gfp-zf-nos*1 3′-UTR RNA was performed according to Yoshizaki et al. (2005), with slight modifications. To prevent chorion hardening, fertilized salmon eggs were incubated in 2 mM L-Glutathione-reduced (Sigma–Aldrich) solution (pH 8.0) at 6°C for 2 hr. A total of 4 nl of the RNA solution supplemented with phenol red (0.05% in working solution, Sigma-Aldrich) was microinjected into the blastodisc at the one-cell stage using an IM-300 microinjector (Narishige, London, UK). The injected eggs were cultured in Hank's solution for 1 day at 6°C, and then transferred to fresh water. GFP expression in embryos was observed at each developmental stage by epifluorescence microscopy. Images were captured with a CCD color camera (AxioCam HRc, Carl Zeiss) connected to a computer equipped with AxioVision 4.1 software (Carl Zeiss). Overall, injection with *gfp-rt-vasa* or *gfp-zf-nos*1 3′-UTR RNA was performed on three batches of fertilized eggs (22–23 eggs per each batch) derived from three different females, and the success rate of microinjection was 74–86% among the batches, as detailed in Supplementary Table S1.

## Abbreviations

*dnd*: *dead end*
dpf: days post-fertilization
dpi: days post-injection
GFP: green fluorescence protein
*ly75/CD205*: *lymphocyte antigen 75*
PGCs: primordial germ cells
