# Supplementary figures and images for: Identification and Migration of Primordial Germ Cells in Atlantic Salmon, Salmo salar: Characterization of Vasa, Dead End, and Lymphocyte Antigen 75 Genes
Source: Mol Reprod Dev. 2013 Feb 5;80(2):118–31. doi: 10.1002/mrd.22142 (PMC3664433; doi:10.1002/mrd.22142)

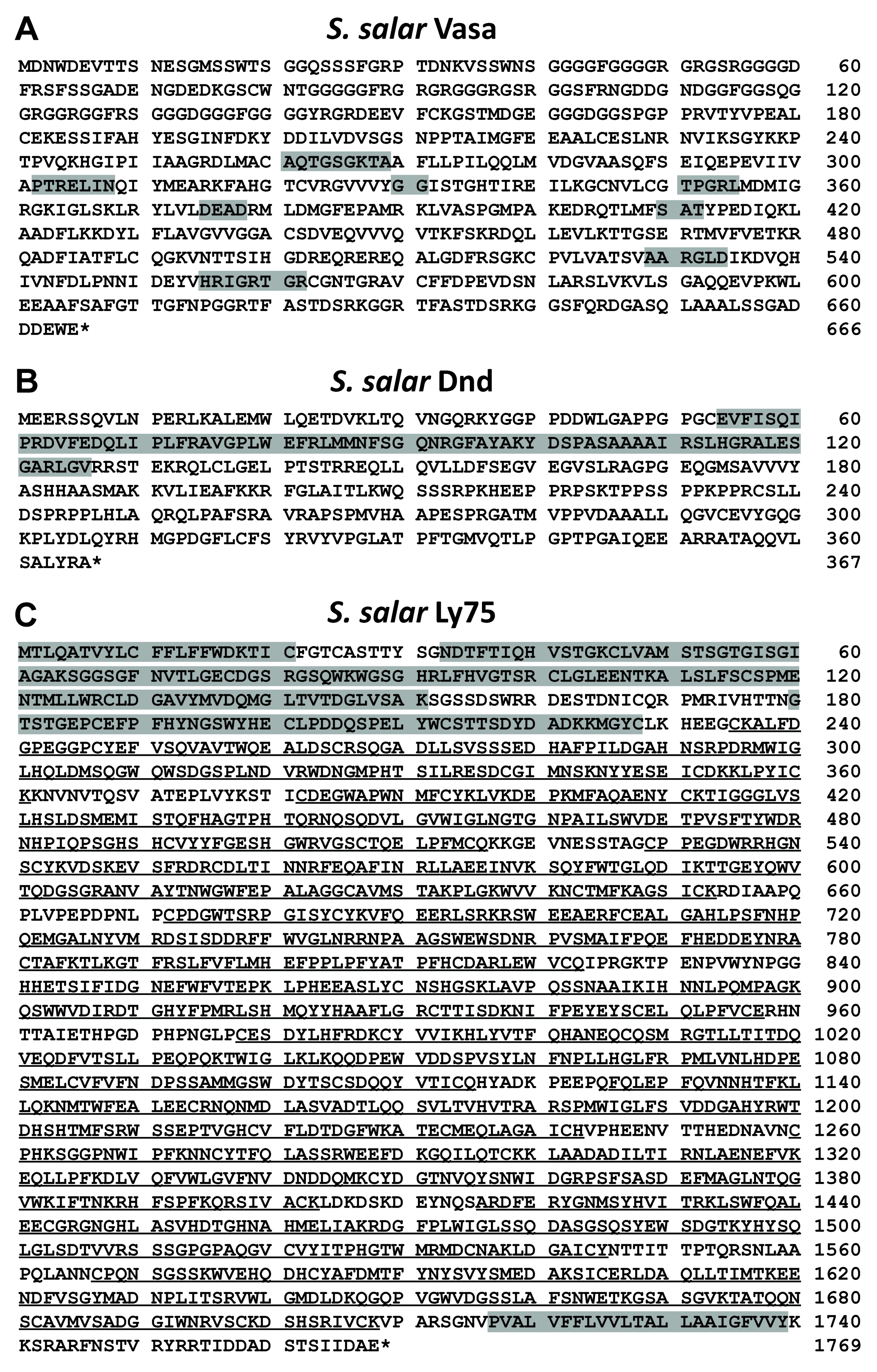

Supplement: Supplementary file 1 [file mrd0080-0118-SD1.tif]
