# Supplementary material for: Identification and Migration of Primordial Germ Cells in Atlantic Salmon, Salmo salar: Characterization of Vasa, Dead End, and Lymphocyte Antigen 75 Genes
Source: Mol Reprod Dev. 2013 Feb 5;80(2):118–31. doi: 10.1002/mrd.22142 (PMC3664433; doi:10.1002/mrd.22142)
Supplement: Supplementary file 2 [file mrd0080-0118-SD2.doc]

**Supplementary Data**

**Fig. S1** Deduced amino acid sequences of Atlantic salmon *vasa*, *dnd*, and *ly75* genes**.**

**A**) Atlantic salmon Vasa amino acid sequences with residue numbers. Eight conserved sequences of DEAD protein family are boxed with grey bars. These sequence data are available from GenBank (JN712912).

**B**) Atlantic salmon Dnd amino acid sequences with residue numbers. The sequence of RNA recognition motif (RRM) domain is boxed with grey bar. These sequence data are available from GenBank (JN712911).

**C**) Atlantic salmon Ly75 amino acid sequences with residue numbers. The sequence of signal peptide (SP), ricin-type beta-trefoil (RICIN/CysR), fibronectin type 2 (FN2), and transmembrane (TM) domains are boxed with grey bars according to the order. Ten C-type lectin (CTLD) domains are underlined with black lines. These sequence data are available from GenBank (JN712913).
