# Supplementary material for: Identification and Migration of Primordial Germ Cells in Atlantic Salmon, Salmo salar: Characterization of Vasa, Dead End, and Lymphocyte Antigen 75 Genes
Source: Mol Reprod Dev. 2013 Feb 5;80(2):118–31. doi: 10.1002/mrd.22142 (PMC3664433; doi:10.1002/mrd.22142)
Supplement: Supplementary file 3 [file mrd0080-0118-SD3.doc]

**Table S1.** Overview of microinjection with *Gfp-rt-vasa* 3’UTR mRNA into Atlantic salmon eggs in this study. The developmental stage is represented by day post-fertilization (dpf) or day post-injection (dpi).

|  | Total eggs* | Fertilized eggs* | Developing embryos (60 dpf)* | Fertilization rate*^, a^ | Survival rate*^, b^ | Injected eggs | GFP (+) eggs at mid-blastula stage (11 dpi) | Embryos with GFP-labeled PGCs at eyed stage (60 dpi) | Injection success rate^†^ |
| --- | --- | --- | --- | --- | --- | --- | --- | --- | --- |
| Female 1 | 423 | 242 | 220 | 57.2% | 90.9% | 23 | 17 | 8 | 73.9% |
| Female 2 | 316 | 250 | 225 | 79.1% | 90.0% | 23 | 19 | 16 | 82.6% |
| Female 3 | 209 | 144 | 116 | 68.9% | 80.6% | 22 | 19 | 8 | 86.4% |

* Note that factors are shown in control group (non-injected embryos).

^a^ Fertilization rate (%) = fertilized eggs / total eggs × 100

^b^  Survival rate (%) = developing embryos (60 dpf) / fertilized eggs × 100

^†^ Injection success rate; GFP-positive eggs at mid-blastula stage (11 dpi) / injected eggs
